# Severed Fingers Reuniting

**Published:** 1893-04

**Authors:** 


### REMARKABLE CASE OF SEVERED FINGERS REUNITING.

Dr. J. M. T. Finney, of Baltimore, relates the following interesting case:

On January 2, 1890, the patient, a machinist by trade, came to the Johns Hopkins Hospital about half-past twelve o'clock, giving the following history. He was a machinist by trade, and was running the engine in the absence of the regular engineer, in a tin shop. He went to work about five o'clock that morning, and a little later, while going about a machine used for chopping blocks of tin, he dropped something, and while stooping down to pick it up his hand slipped under the knife and the ends of the middle and ring fingers were cut off. The middle finger was cut off just beyond the last joint. The joint was opened. The ring finger was cut off just above the root of the nail. This occurred, the man said, about half-past five o'clock. He wrapped up the stumps and went home, where his wife covered the wounds with beeswax. He arrived at the hospital at the time previously stated. I asked him where the stumps of the fingers were and he produced them wrapped up in a piece of newspaper. They were very cold, almost frozen. I placed them in a basin of warm water, using no antiseptic, because bichloride or carbolic acid might cause a layer of coagulation necrosis and prevent union. I scrubbed up the stumps of the fingers with a 1-2,000 warm bichloride solution, then I carefully rinsed them off in warm water. This process consumed at least half an hour. Then I took a shaving off the ends of the fingers, so as to have a perfectly fresh surface. The stumps were treated in the same manner. The bone was scraped. I sewed them on, using four stiches in each case. I then applied strips of crepe lisse with collodion the whole length of the fingers on each side. These held the several portions in exact apposition. Then I used other strips around the fingers, binding them together, and then applied a palmar splint and used a large absorbent dressing. He came back in a week, and when the dressing was removed the fingers looked very well. I reapplied the dressing and told him to report in another week. Dr. Brockway saw the case on his return at the end of the second week. He took out the stitches and removed the dressing and said that there was no doubt that the fingers had united and that the man seemed to have sensation at the ends of the fingers, although he thought that this sensation might have been transmitted. The man then disappeared entirely from view. He returned about a month ago with an injury to his other hand. It is difficult to say at first sight which hand was injured. There is a slight motion in the joint which was opened, and the sensation in the fingers is perfect.

